# Combined GPS/GLONASS Precise Point Positioning with Fixed GPS Ambiguities

**DOI:** 10.3390/s140917530

**Published:** 2014-09-18

**Authors:** Lin Pan, Changsheng Cai, Rock Santerre, Jianjun Zhu

**Affiliations:** 1 School of Geosciences and Info-Physics, Central South University, Changsha 410083, China; E-Mails: panlin@mail.csu.edu.cn (L.P.); zjj@mail.csu.edu.cn (J.Z.); 2 Département des Sciences Géomatiques, Université Laval, Québec G1V 0A6, Canada; E-Mail: Rock.Santerre@scg.ulaval.ca

**Keywords:** GPS, GLONASS, precise point positioning, integer ambiguity resolution

## Abstract

Precise point positioning (PPP) technology is mostly implemented with an ambiguity-float solution. Its performance may be further improved by performing ambiguity-fixed resolution. Currently, the PPP integer ambiguity resolutions (IARs) are mainly based on GPS-only measurements. The integration of GPS and GLONASS can speed up the convergence and increase the accuracy of float ambiguity estimates, which contributes to enhancing the success rate and reliability of fixing ambiguities. This paper presents an approach of combined GPS/GLONASS PPP with fixed GPS ambiguities (GGPPP-FGA) in which GPS ambiguities are fixed into integers, while all GLONASS ambiguities are kept as float values. An improved minimum constellation method (MCM) is proposed to enhance the efficiency of GPS ambiguity fixing. Datasets from 20 globally distributed stations on two consecutive days are employed to investigate the performance of the GGPPP-FGA, including the positioning accuracy, convergence time and the time to first fix (TTFF). All datasets are processed for a time span of three hours in three scenarios, *i.e.*, the GPS ambiguity-float solution, the GPS ambiguity-fixed resolution and the GGPPP-FGA resolution. The results indicate that the performance of the GPS ambiguity-fixed resolutions is significantly better than that of the GPS ambiguity-float solutions. In addition, the GGPPP-FGA improves the positioning accuracy by 38%, 25% and 44% and reduces the convergence time by 36%, 36% and 29% in the east, north and up coordinate components over the GPS-only ambiguity-fixed resolutions, respectively. Moreover, the TTFF is reduced by 27% after adding GLONASS observations. Wilcoxon rank sum tests and chi-square two-sample tests are made to examine the significance of the improvement on the positioning accuracy, convergence time and TTFF.

## 1. Introduction

With the use of precise satellite orbit and clock products, precise point positioning (PPP) technology can provide centimeter-level or even millimeter-level positioning accuracy using un-differenced carrier phase observations [1]. As the fractional cycle biases (FCB) that are contained in the carrier phase observations cannot be separated from the integer ambiguities [2], the ambiguities are usually estimated as float values, which restricts the further enhancement of PPP performance. To obtain ambiguity-fixed PPP resolutions, proper handling of the FCB is a key issue. There are presently two main ways to solve this issue. One way is to estimate the FCB using a network of reference stations [2–5]. The other way is the integer recoverable clock (IRC) method in which the satellite FCB are assimilated into satellite clock corrections when generating the clock products [6,7]. Both methods are implemented using GPS-only measurements. Although significant progress has been made regarding the PPP integer ambiguity resolutions (IAR) in recent years, the PPP still needs a convergence time of typically 20 min [8], and the positioning accuracy remains on a level of 1–3 cm [9,10].

An effective way of improving PPP performance is to integrate GPS and GLONASS. A full GLONASS constellation consisting of 24 operational satellites has been revitalized to date [11]. The quality of GLONASS precise orbit and clock products routinely provided by several IGS (International GNSS Service) analysis centers, e.g., ESA/ESOC (European Space Agency/European Space Operations Centers, Germany) and IAC (Information-Analytical Center, Russia), are gradually improved, as more than 180 IGS stations can continuously track GPS and GLONASS signals [12]. The integration of GLONASS and GPS has been confirmed to be able to improve the ambiguity-float PPP performance by several researchers in terms of availability, reliability, accuracy and convergence time of position solutions [13–16]. According to [17], the accuracy of float ambiguities has a large impact on the successful rate and reliability of PPP IAR. Thus, an improvement on the success rate and reliability of the IAR can be expected, since the integration of GPS and GLONASS can enhance the quality of the float ambiguity estimates. Jokinen *et al.* [18] implemented the GLONASS-aided GPS ambiguity-fixed PPP for the first time. Unfortunately, only slight improvements for the positioning accuracy and convergence time were achieved in their results, likely because a much smaller weight was assigned to GLONASS measurements. Li and Zhang [19] investigated the contribution of GLONASS observations to the time to first fix (TTFF) in the GPS ambiguity-fixed PPP resolutions, but the positioning accuracy was not assessed.

This paper presents an approach of combined GPS/GLONASS PPP with fixed GPS ambiguities (GGPPP-FGA) in which GPS ambiguities are fixed into integers, while all GLONASS ambiguities are kept as float values. An improved minimum constellation method is proposed for the purpose of increasing computation efficiency. Datasets from 20 globally distributed IGS stations on two consecutive days are employed to fully assess the performance of the GGPPP-FGA with a comparison to GPS ambiguity-fixed resolutions and GPS ambiguity-float solutions. Further, Wilcoxon rank sum tests [20] and chi-square two-sample tests [21] are made to examine the significance of the performance differences between the three sets of solutions.

## 2. CNES Satellite Orbit and Clock Products

The precise satellite orbit and clock corrections provided by CNES (Centre National d'Études Spatiales, France) are employed for GGPPP-FGA processing. The FCB of GPS carrier-phase measurements on the satellite end has been assimilated into the CNES satellite clock corrections when the clock products are generated by a carrier-phase-based network solution [17]. By contrast, the GLONASS FCB on the satellite end cannot be completely assimilated into the satellite clock corrections due to the adoption of a code-phase-based network solution [17]. The root mean square (RMS) of GPS satellite orbit three-dimensional (3D) errors is better than 5.0 cm, and the standard deviation (STD) of GPS clock corrections is 0.12 ns, namely 3.6 cm. Such an accuracy of orbit and clock products is sufficient for carrying out GPS IAR research according to [17]. Since the accuracy of the CNES GLONASS satellite orbit and clock products is not publicly available so far [18], it is assessed in this section by comparing with the products from other analysis centers.

The data analysis centers of ESA/ESOC and IAC provide post-processed GLONASS satellite orbit and clock products. Precise products from both centers are taken as references to analyze the accuracy of the CNES GLONASS satellite orbit and clock corrections, which are generated in real time. The final satellite orbit and clock products from both ESA/ESOC and IAC have a sampling interval of 15 min and 30 s, respectively, whereas the CNES satellite orbit and clock corrections are recorded at 5 min and 5 s, respectively. For consistency, the CNES products are re-sampled to match with the ESA/ESOC and IAC products.

**Figure 1.**
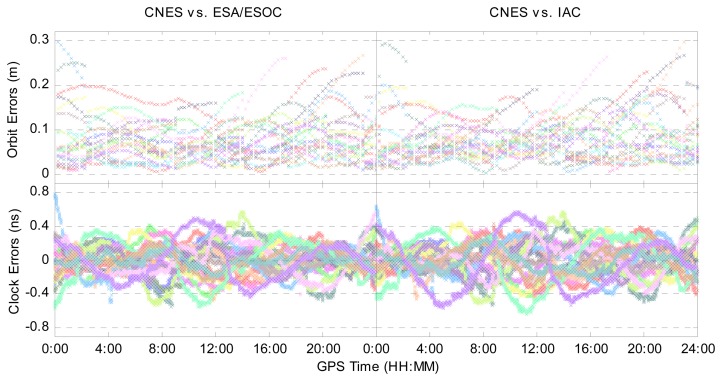
Errors of CNES (Centre National d'Études Spatiales) GLONASS satellite orbit and clock corrections with respect to the ESA/ESOC (European Space Agency/European Space Operations Centers) and IAC (Information-Analytical Center) products on 1 February 2014.

Figure 1 shows the errors of the CNES GLONASS satellite orbit and clock corrections on 1 February 2014 with respect to the ESA/ESOC and IAC ones. Different colors represent different GLONASS satellites. In the bottom panels of Figure 1, a common clock bias with respect to all GLONASS satellites and a constant clock bias that is different from different GLONASS satellites have been removed, since they will be absorbed into the receiver clock and ambiguity items, respectively, in the parameter estimation process. It is clearly seen that most of the time, the GLONASS satellite orbit errors vary in a range of 20 cm, while clock errors vary in a range of 0.4 ns. The RMSs of GLONASS satellite orbit 3D errors and clock errors with respect to ESA/ESOC and IAC products are shown in Table 1. The results indicate that the average RMSs of orbit errors and clock errors are 7.9 cm and 0.14 ns, respectively. For comparison, Table 1 also provides the average RMSs of GPS orbit errors and clock errors, respectively, using the corresponding GPS products on the same day. It is clear that the accuracy of the CNES GLONASS precise products is poorer than its GPS ones.

## 3. Approach of Combined GPS/GLONASS PPP with Fixed GPS Ambiguities

The functional model, stochastic model and the error handling strategy for combined GPS/GLONASS ambiguity-float PPP have been discussed in [15]. Taking the FCB into account in the observation equations, the code and carrier-phase observation models on *L_1_* and *L_2_* frequencies for a GPS or GLONASS satellite can be expressed as:

(1) $$P_{i}=\rho +c(dt-dT)+d_{\text{orb}}+d_{\text{trop}}+d_{\text{ion}/L_{i}}+{ɛ}_{P_{i}}$$

(2) $$\Phi_{i}=\rho +c(dt-dT)+d_{\text{orb}}+d_{\text{trop}}-d_{\text{ion}/L_{i}}+\lambda_{i}(N_{i}+b_{i}-b^{i})+{ɛ}_{\Phi_{i}}$$

where *P_i_* is the measured code on *L_i_* in meters, Φ*_i_* is the measured carrier-phase on *L_i_* in meters, *ρ* is the geometric range in meters, *c* is the speed of light in vacuum in meters per second, *d_t_* is the receiver clock offset in seconds, *dT* is the satellite clock offset in seconds, *d_orb_* is the satellite orbit error in meters, *d_trop_* is the tropospheric delay in meters, *d_ion/L_i__* is the ionospheric delay on *L_i_* in meters, *λ_i_* is the wavelength on *L_i_* in meters per cycle, *N_i_* is the phase ambiguity on *L_i_* in cycles, *b_i_* is the receiver FCB on *L_i_* in cycles, *b^i^* is the satellite FCB on *L_i_* in cycles and *ε* includes the multipath effect and measurement noise in meters.

As the CNES satellite orbit and clock products refer to precise code measurements of *P_1_* and *P_2_*, the differential code biases (DCB) between *C_1_* and *P_1_* or between *C_2_* and *P_2_* must be corrected when the civil code measurements *C_1_* or *C_2_* are used. The DCB corrections provided monthly by CODE (Center for Orbit Determination in Europe, Switzerland) are used to remove the DCB from measurements in this study, since the value could be up to 1.2 m [22].

The single-difference (SD) observations between GPS satellites are employed to remove the GPS FCB on the receiver end. The GPS satellite at the highest elevation angles is chosen as the base satellite when forming the SD observations. After applying the precise satellite orbit and clock corrections, the SD ionosphere-free observation models can be expressed as follows:

(3) $$\Delta P_{IF}^{k,j}=P_{IF}^{k}-P_{IF}^{j}=\Delta \rho^{k,j}+\Delta d_{trop}^{k,j}+\Delta {ɛ}_{P_{IF}}^{k,j}$$

(4) $$\Delta \Phi_{IF}^{k,j}=\Phi_{IF}^{k}-\Phi_{IF}^{j}=\Delta \rho^{k,j}+\Delta d_{\text{trop}}^{k,j}+\lambda_{1}(\Delta N_{IF}^{k,j}-\Delta b_{IF}^{k,j})+\Delta {ɛ}_{\Phi_{IF}}^{k,j}$$

where the index *j* represents one GPS base satellite and the index *k* represents another GPS satellite. *P_IF_* and Φ*_IF_* are the ionosphere-free combined code and carrier-phase observations in meters, respectively. *ΔN_IF_* and $\Delta b_{IF}^{kj}$ are the SD ionosphere-free ambiguity and satellite FCB in cycles, respectively. They can be expressed as:

(5) $$\Delta N_{IF}^{k,j}=\frac{f_{1}^{2}\Delta N_{1}^{k,j}-f_{1}f_{2}\Delta N_{2}^{k,j}}{f_{1}^{2}-f_{2}^{2}}$$

(6) $$\Delta b_{IF}^{k,j}=\frac{f_{1}^{2}\Delta b_{1}^{k,j}-f_{1}f_{2}\Delta b_{2}^{k,j}}{f_{1}^{2}-f_{2}^{2}}$$

where *f_1_* and *f_2_* are the GPS carrier-phase frequency on *L_1_* and *L_2_* in Hertz, respectively. *ΔN_1_* and *ΔN_2_* are the SD ambiguities on *L_1_* and *L_2_* in cycles, respectively. $\Delta b_{1}^{kj}$ and $\Delta b_{2}^{kj}$ are the SD satellite FCB on *L_1_* and *L_2_* in cycles, respectively.

As can be seen from Equation (5), the SD ionosphere-free ambiguity *ΔN_IF_* is not an integer, but it can be decomposed into an integer SD wide-lane ambiguity (*ΔN_WL_* in cycles) and an integer SD narrow-lane ambiguity (*ΔN_NL_* in cycles). The decomposition can be described below:

(7) $$\Delta N_{IF}^{k,j}=\frac{f_{1}f_{2}}{f_{1}^{2}-f_{2}^{2}}\Delta N_{WL}^{k,j}+\frac{f_{1}}{f_{1}+f_{2}}\Delta N_{NL}^{k,j}$$

In this study, the GPS IAR is carried out in two stages: Fixing the wide-lane ambiguities in the first stage and fixing the narrow-lane ambiguities in the second stage.

### 3.1. GPS Wide-Lane Ambiguity Fixing

GPS wide-lane ambiguity float values are estimated using the Melbourne-Wübbena combination [23,24], as shown in Equations (8) and (9):

(8) $$\Delta \Phi_{MW}^{k,j}=\frac{f_{1}\cdot \Delta \Phi_{1}^{k,j}-f_{2}\cdot \Delta \Phi_{2}^{k,j}}{f_{1}-f_{2}}-\frac{f_{1}\cdot \Delta P_{1}^{k,j}+f_{2}\cdot \Delta P_{2}^{k,j}}{f_{1}+f_{2}}$$

(9) $$\Delta N_{WL}^{k,j}=\Delta \Phi_{MW}^{k,j}/\lambda_{WL}$$

where *λ_WL_* is the wavelength of GPS wide-lane ambiguity in meters per cycle and *λ_WL_*=*c*/(*f*_1_-*f*_2_)≈0.86 *m*, $\Delta N_{WL}^{kj}$, is the GPS SD wide-lane ambiguity in cycles and $\Delta N_{WL}^{kj}=\Delta N_{1}^{kj}-\Delta N_{2}^{kj}$.

The wide-lane FCB corrections on the satellite end are applied to recover the integer properties of GPS wide-lane ambiguities before implementing the wide-lane IAR. CNES provides a daily update of GPS wide-lane FCB corrections on the satellite end [17]. The wide-lane IAR is achieved by rounding the wide-lane ambiguity float values to the nearest integers, because the wavelength of the wide-lane ambiguity is as long as 0.86 m. Despite the long wavelength, it is possible that the higher noise of the wide-lane ambiguity estimates causes the wrong fixation at some epochs. In order to increase the reliability, the following smoothing operation is applied:

(10) $$<\Delta N_{WL}{>}_{i}=<\Delta N_{WL}{>}_{i-1}+\frac{1}{i}(\Delta N_{WLi}-<\Delta N_{WL}{>}_{i-1})$$

where <*ΔN_WL_*>*_i_* is the mean value of wide-lane ambiguities from epochs *1*-th to *i*-th and *ΔN_WLi_* is the wide-lane ambiguity at the *i*-th epoch. The cycle-slip detection is carried out before the smoothing operation. If a cycle-slip is detected, the smoothing operation is restarted from the epoch that the cycle slip occurs. The TurboEdit algorithm [25] and the phase ionospheric residual method [26] are jointly used to detect cycle-slips.

### 3.2. GPS Narrow-Lane Ambiguity Fixing

The receiver FCB included in GPS narrow-lane ambiguities have been removed by the SD operation. The GPS satellite FCB have been assimilated into the CNES satellite clock corrections. Hence, the integer properties of GPS narrow-lane ambiguities can be recovered after applying the satellite clock corrections [17]. According to Equation (7), the float narrow-lane ambiguity can be derived by deducting the fixed wide-lane ambiguity from the float ionosphere-free ambiguity. Since the success rate of fixing wide-lane ambiguity is pretty high due to its long wavelength, the accuracy of float narrow-lane ambiguity estimates is mainly affected by the quality of float ionosphere-free ambiguities.

The least-squares ambiguity de-correlation adjustment (LAMBDA) method [27] is employed to implement the GPS narrow-lane IAR. At least four float narrow-lane ambiguities are required at each epoch before the LAMBDA method can be used [18,19]. When more than four float narrow-lane ambiguities are available, the minimum constellation method (MCM) [28,29] is adopted to carry out the IAR. The purpose of the MCM is to find out an optimal float ambiguity combination for ambiguity fixing. In order to ensure the reliability of narrow-lane IAR, not all GPS narrow-lane ambiguities are fixed to integers. The MCM attempts to calculate the IAR with all possible combinations of float narrow-lane ambiguities, but each combination contains at least four float ambiguities. Thus, it is possible that all float ambiguities are selected as a combination for ambiguity fixing. For example, if there are six float narrow-lane ambiguities available at a certain epoch, IAR can be made for one six-satellite group, six five-satellite groups and fifteen four-satellite groups. It is easily understood that the efficiency of the MCM algorithm is pretty low, since all possible combinations need to be tested.

An improved MCM (IMCM) algorithm is proposed to cut down the amount of combinations to be tested in this study. In this IMCM algorithm, the float ambiguities at satellite elevation angles lower than 10° are firstly removed from the ambiguity candidates, due to their larger noise and residual errors. All the float narrow-lane ambiguity candidates are required to have already converged before they are fixed. For a certain GPS satellite, if the STD of float narrow-lane ambiguities is smaller than 0.1 cycles within five consecutive epochs, the float narrow-lane ambiguities of this satellite are considered to have converged in this study. The values of “0.1” cycles and “five” epochs are empirically determined after considering a compromise between reliability and efficiency. Once there are a minimum of four float narrow-lane ambiguities that have converged at an epoch, they will be firstly fixed using the LAMBDA method in which the narrow-lane ambiguity validation is carried out by the ratio test with a ratio value setting of 2.0 [30]. If the ratio test passes successfully, the four float narrow-lane ambiguities are fixed into integers. When ambiguities from over four satellites have already converged, the IMCM is performed to pick out an optimal float ambiguity combination for ambiguity fixing. If the float narrow-lane ambiguities from a certain satellite have been fixed to the same integer for ten consecutive epochs using the IMCM algorithm, the narrow-lane ambiguity of the satellite will be included into the ambiguity combination for IAR. As a result, the amount of ambiguity combinations to be tested will be gradually decreased. Using the improved MCM algorithm, the computation efficiency is considerably improved, which will be demonstrated through a case study in the next section.

For GLONASS, there exist inter-frequency biases in code and phase measurements [18,19], caused by the frequency division multiple access (FDMA) technique. As a result, the SD operation between satellites cannot effectively remove the FCB on the receiver end. For this reason, the above GPS IAR approach does not apply to GLONASS. Instead of IAR, GLONASS ambiguities are kept as float solutions. As stated before, the accuracy of float narrow-lane ambiguity estimates is quite dependent on the quality of float ionosphere-free ambiguities. The addition of GLONASS observations is expected to improve the successful rate and reliability of GPS IAR, since it can help improve the accuracy of float ionosphere-free ambiguities [18].

## 4. Results and Discussion

### 4.1. Data Acquisition

**Figure 2.**
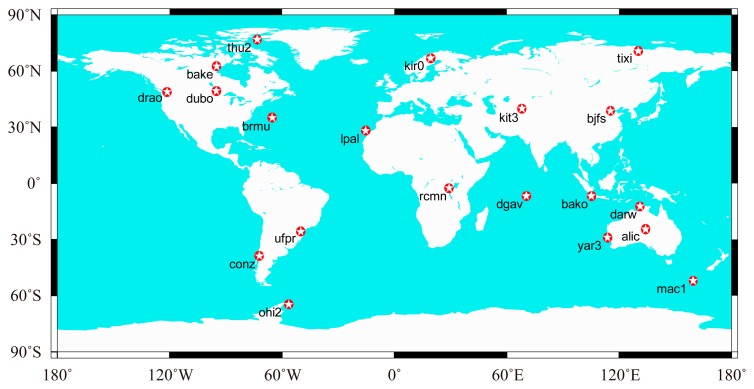
Geographical distribution of 20 IGS (International GNSS Service) stations.

Datasets collected from 20 globally distributed IGS stations from 1–2 February 2014, are used to assess the performance of the GGPPP-FGA. The distribution of stations is shown in Figure 2. All observations have a sampling rate of 30 s with a satellite elevation mask angle of 7°. The GPS and GLONASS satellite orbit and clock products provided by CNES are used for PPP processing. Although the PPP is performed in a post-processing mode, it has a good indication of the real-time PPP performance, since the CNES products are generated in real time.

**Figure 3.**
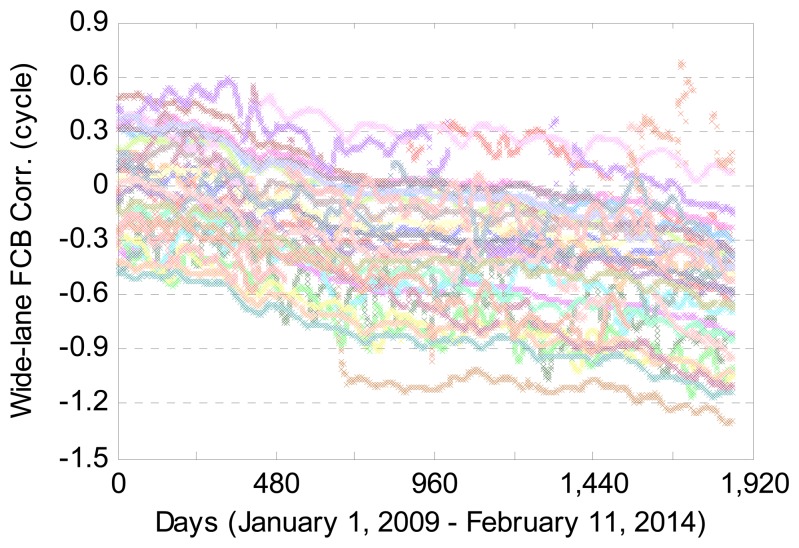
GPS wide-lane fractional cycle biases (FCB) corrections on the satellite end for 1863 days from 1 January 2009 to 11 February 2014.

**Figure 4.**
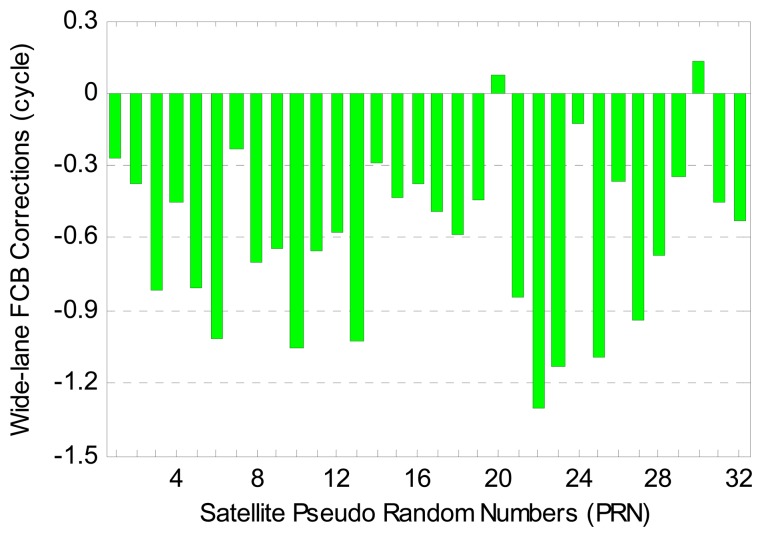
GPS wide-lane FCB corrections on the satellite end on 1 February 2014.

Figure 3 shows the GPS wide-lane FCB corrections on the satellite end for 1863 days from 1 January 2009 to 11 February 2014. The corrections are provided daily by CNES. Different colors represent different GPS satellites. As can be seen, the wide-lane FCB corrections have a good stability over time. As a whole, the FCB corrections of all GPS satellites have a decreasing trend with time. Figure 4 provides the wide-lane FCB corrections for all 32 GPS satellites on 1 February 2014. It is observed that the FCB corrections for most GPS satellites exceed −0.5 cycles, which suggests that the correction of the FCB is quite necessary in order to achieve the wide-lane IAR.

### 4.2. Results and Analysis

For the purpose of comparison, three scenarios are used for PPP processing. The first one is the GPS ambiguity-float PPP solution using un-differenced GPS-only observations. The second one is GPS ambiguity-fixed PPP resolution using SD GPS-only observations. The third one is the GGPPP-FGA resolution using SD GPS observations and un-differenced GLONASS observations. In the last two scenarios, only GPS ambiguities are fixed based on the IMCM algorithm. In this study, the STD values of GPS code and carrier-phase measurements are set to 0.3 m and 0.002 m, respectively, while the GLONASS ones are set to 0.6 m and 0.002 m, respectively [15].

**Figure 5.**
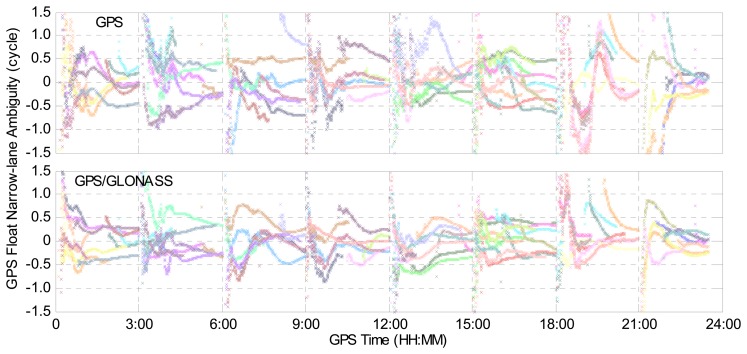
GPS narrow-lane ambiguity-float values for GPS-only and combined GPS/GLONASS precise point positioning at IGS station BJFS on 1 February 2014.

In order to compare the GPS narrow-lane ambiguity-float estimates for GPS-only and combined GPS/GLONASS PPP cases, the float narrow-lane ambiguities for different GPS satellites are plotted in Figure 5, using the observations at IGS station BJFS on 1 February 2014. Different colors represent different GPS satellites. In this section, the 24-hour dataset is divided into eight sessions with a session length of three hours. For the convenience of display, the same integer is subtracted from the float narrow-lane ambiguities for the same GPS satellite in the two cases. Hence, all of the narrow-lane ambiguities approach zero in Figure 5. It can be clearly seen that the float narrow-lane ambiguities in the GPS/GLONASS case can reach stable values more quickly than those in the GPS-only case, which demonstrates that the addition of GLONASS improves the performance of GPS narrow-lane float ambiguity estimates.

**Figure 6.**
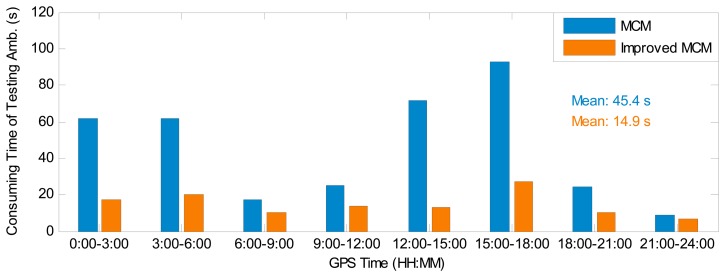
Computation time of testing GPS float narrow-lane ambiguity combinations in minimum constellation method (MCM) and improved MCM (IMCM) for combined GPS/GLONASS precise point positioning with fixed GPS ambiguities (GGPPP-FGA) at IGS station BJFS on 1 February 2014.

As stated before, the MCM is used to choose the optimal float narrow-lane ambiguity combination for IAR. To validate the improved computation efficiency for the IMCM in GGPPP-FGA, Figure 6 presents the computation time of testing GPS float narrow-lane ambiguity combinations for the MCM and IMCM algorithms in eight sessions using the observations at station BJFS on 1 February 2014. The consuming time in each session is an accumulative value of the testing time at each epoch, based on a computer configuration of a 2.9-GHz Intel Pentium G2020 CPU and 4 GB of random-access memory. The results clearly illustrate that the consuming time of testing ambiguities in the IMCM is much smaller than that in the MCM algorithm. The average time of testing GPS float narrow-lane ambiguity combinations for all eight sessions is also provided in Figure 6. The statistical results indicate that the consuming time to obtain an optimal combination is reduced by 67% from 45.4 s to 14.9 s when adopting the IMCM algorithm. When processing the observations at IGS stations KIR0 and RCMN on 1 February 2014, with the IMCM algorithm, the consuming time is reduced by 59% and 64%, respectively. In conclusion, the IMCM indeed significantly improves the computation efficiency in the process of the narrow-lane ambiguity fixing.

**Figure 7.**
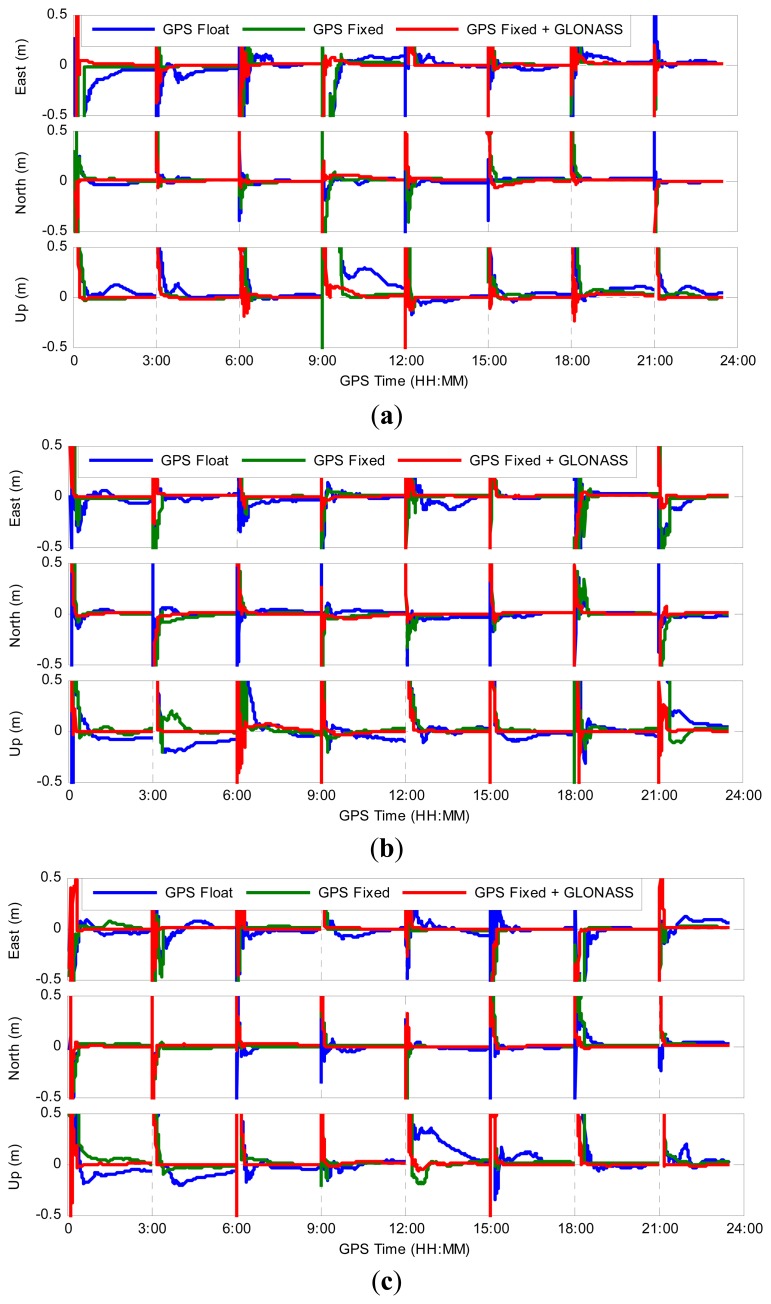
Positioning errors for GPS ambiguity-float PPP, GPS ambiguity-fixed PPP and GGPPP-FGA using observations on 1 February 2014. The IGS datasets are collected from (**a**) a high latitude station KIR0, (**b**) a mid-latitude station BJFS, and (**c**) a low latitude station RCMN.

Figure 7 shows the positioning errors for three scenarios, *i.e.*, GPS-only ambiguity-float PPP, GPS-only ambiguity-fixed PPP and GGPPP-FGA processing, for all eight three-hour sessions on 1 February 2014. As representatives, the processing results at three stations, KIR0, BJFS and RCMN, are displayed in Figure 7. The three stations are distributed at high, middle and low latitude regions, respectively. From Figure 7, it can be clearly seen how the position filter converges in east, north and up directions. In most cases, the positioning errors for GPS ambiguity-fixed PPP are dramatically reduced immediately once the integer ambiguities are fixed in comparison to GPS ambiguity-float solutions. However, for some sessions, this is not the case, e.g., 3:00–6:00 at BJFS, 21:00–24:00 at BJFS, 0:00–3:00 at RCMN and 12:00–15:00 at RCMN. In these sessions, the positioning errors for GPS ambiguity-fixed PPP are not significantly decreased and even larger than 1 dm after GPS ambiguities are fixed. The reason is that the ambiguities of part GPS satellites may be fixed into wrong integers, due to the poorer accuracy of GPS ambiguity-float estimates. After adding GLONASS observations, the GGPPP-FGA achieves better results in all of these sessions. It is clear that the GGPPP-FGA achieves higher positioning accuracy and a shorter convergence time in almost all sessions, in comparison to GPS ambiguity-float or ambiguity-fixed PPP solutions.

**Figure 8.**
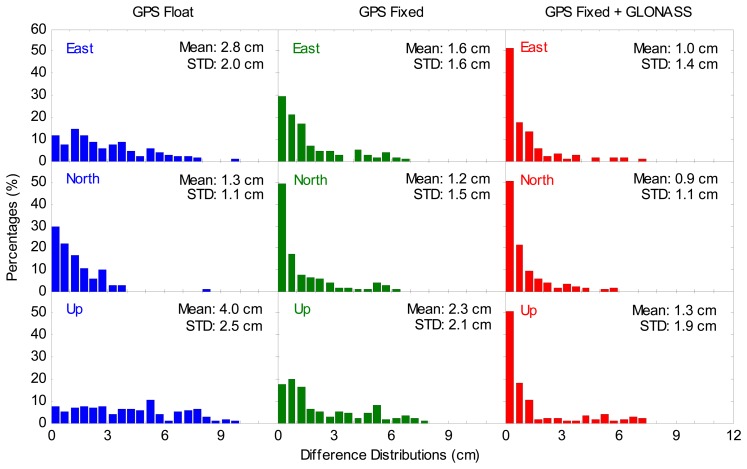
Distributions of positioning errors for GPS ambiguity-float PPP, GPS ambiguity-fixed PPP and GGPPP-FGA processing in eight sessions using datasets collected at 20 IGS stations on two consecutive days. The distributions in the east, north and up components are displayed in the **top**, **middle** and **bottom** panels, respectively.

In order to assess the positioning accuracy, the positioning errors for all 320 sessions at 20 IGS stations on two days are plotted in Figure 8. Each error value refers to the RMS of the position errors for the last 20 min of each session. Figure 8 clearly illustrates that the GGPPP-FGA case accounts for the least percent of errors larger than 3 cm, and the GPS ambiguity-fixed PPP case follows. The average positioning errors for all sessions are also provided in Figure 8. According to the statistical results, the improvement of GPS ambiguity-fixed PPP on the average positioning accuracy is 43%, 8% and 43% over GPS ambiguity-float PPP in the east, north and up coordinate components, respectively. The reason for the small improvement in the north component is that the north position component is already of good quality, even for ambiguity-float solutions [15], which is related to the GPS constellation configuration. See also [31] for a comparison about fixed and float carrier phase ambiguity solutions. Furthermore, the improvement of the GGPPP-FGA over the GPS ambiguity-fixed PPP is 38%, 25% and 44% in three coordinate components, respectively. The average 3D positioning accuracy for the GPS ambiguity-float PPP, GPS ambiguity-fixed PPP and GGPPP-FGA is 5.1 cm, 3.1 cm and 1.9 cm, respectively. Furthermore, using the CNES satellite orbit and clock products, Laurichesse [17] and Jokinen *et al.* [18] implemented the GPS ambiguity-fixed PPP at a 3D accuracy of approximately 4 cm. The GPS ambiguity-fixed PPP accuracy from our statistical results is comparable to theirs.

In order to examine the significance of the accuracy improvement, Wilcoxon rank sum tests were made to indicate whether two sets of solutions obey the same distribution or not. The Wilcoxon rank sum tests were first made between GGPPP-FGA resolutions and GPS ambiguity-fixed resolutions in the east, north and up components, respectively. In the Wilcoxon rank sum test, the null hypothesis is H0: Two sets of solutions come from a common distribution. The alternative hypothesis is Ha: Two sets of solutions come from a different distribution. Since the number of solutions is 320 for each set, the Wilcoxon rank sum statistic values (*W*) are close to a normal distribution. When the significance level is selected as 5%, their respective critical values are both 1.96. If *W* < 1.96, the null hypothesis should be accepted. Otherwise, the null hypothesis should be rejected. The Wilcoxon rank sum statistic values are 5.99, 2.24 and 8.99 in three coordinate components, respectively. Apparently, all of the Wilcoxon rank sum statistic values are larger than the critical value. The null hypotheses are therefore rejected, suggesting that the GGPPP-FGA resolutions and GPS ambiguity-fixed resolutions follow different distributions for all three components. Similar tests were made between GPS ambiguity-fixed resolutions and GPS ambiguity-float solutions, and the results indicate that they also follow different distributions.

To further examine the significance of the error-distribution percentage difference for the three sets of solutions presented in Figure 8, the chi-square two-sample tests were made between GGPPP-FGA resolutions and GPS ambiguity-fixed resolutions and between GPS ambiguity-fixed resolutions and GPS ambiguity-float solutions, respectively. In the chi-square two-sample test, the null hypothesis is H0: two sets of error-distribution percentages are the same. The alternative hypothesis is Ha: two sets of error-distribution percentages are different. The significance level is selected as 5%. With the confidence coefficient of 95% and the computed degree of freedom (DOF), if $\chi^{2}<\chi_{0.05,\text{DOF}}^{2}$, the null hypothesis should be accepted. Otherwise, the null hypothesis should be rejected. For any two sets of solutions, the chi-square two-sample tests were made in the east, north and up coordinate components, respectively. Table 2 provides the chi-square statistic values for all chi-square two-sample tests. Apparently, all of the chi-square statistic values (*χ^2^*) are larger than their respective critical values $\left(\chi_{0.05,\text{DOF}}^{2}\right)$. The null hypotheses are therefore rejected. This clearly suggests that the error-distribution percentages of the GGPPP-FGA resolutions, GPS ambiguity-fixed resolutions and GPS ambiguity-float solutions are different.

The Wilcoxon rank sum and chi-square two-sample test results indicate that the three sets of solutions obey different distributions and that their error-distribution percentages are also different, which means that their accuracies are different. Thus, the solutions with smaller mean values and STDs apparently have higher accuracy. According to the mean values and STDs shown in Figure 8, it is statistically concluded that the accuracy improvement of the GGPPP-FGA over the GPS ambiguity-fixed PPP is significant. The accuracy improvement of GPS ambiguity-fixed PPP over the GPS ambiguity-float PPP is also significant, except for the north coordinate component.

**Figure 9.**
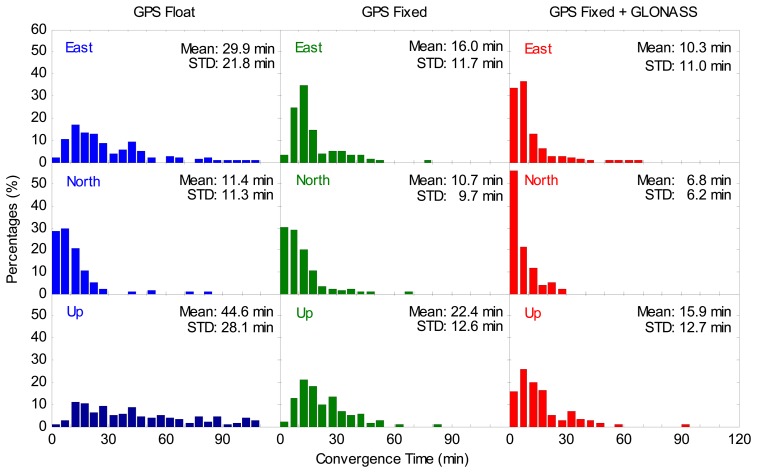
Distributions of convergence time for GPS ambiguity-float PPP, GPS ambiguity-fixed PPP and GGPPP-FGA processing in eight sessions using datasets collected at 20 IGS stations on two consecutive days. The distributions in the east, north and up components are displayed in the **top**, **middle** and **bottom** panels, respectively. The criterion of convergence is defined when the positioning errors reach 0.1 m and keep within 0.1 m.

Figure 9 depicts the distributions of the convergence time. In this study, the position filter is considered to have converged when the positioning errors reach 0.1 m and keeps within 0.1 m [19] in the east, north and up coordinate components, respectively. The percentages of the convergence time shorter than 10 min in the GGPPP-FGA case are significantly larger than those in the GPS ambiguity-fixed case. Figure 9 also shows the average convergence time for all sessions. According to this statistics, the improvement for the GPS ambiguity-fixed PPP on the average convergence time is 47%, 6% and 50% over GPS ambiguity-float PPP in the east, north and up coordinate components, respectively. The average convergence time for the GGPPP-FGA has an improvement of 36%, 36% and 29% over the GPS ambiguity-fixed PPP in three coordinate components, respectively. To examine the significance of the improvement on convergence time, similar statistical tests were made. Statistical results indicate that all of the Wilcoxon rank sum statistic values and chi-square statistic values are larger than their respective critical values, respectively, except for the north component test between the GPS ambiguity-fixed resolutions and the GPS ambiguity-float solutions. The test results indicate that the improvement of convergence time for the GGPPP-FGA over the GPS ambiguity-fixed PPP is significant. The improvement of convergence time for the GPS ambiguity-fixed PPP over the GPS ambiguity-float PPP is also significant, except for the north coordinate component.

**Figure 10.**
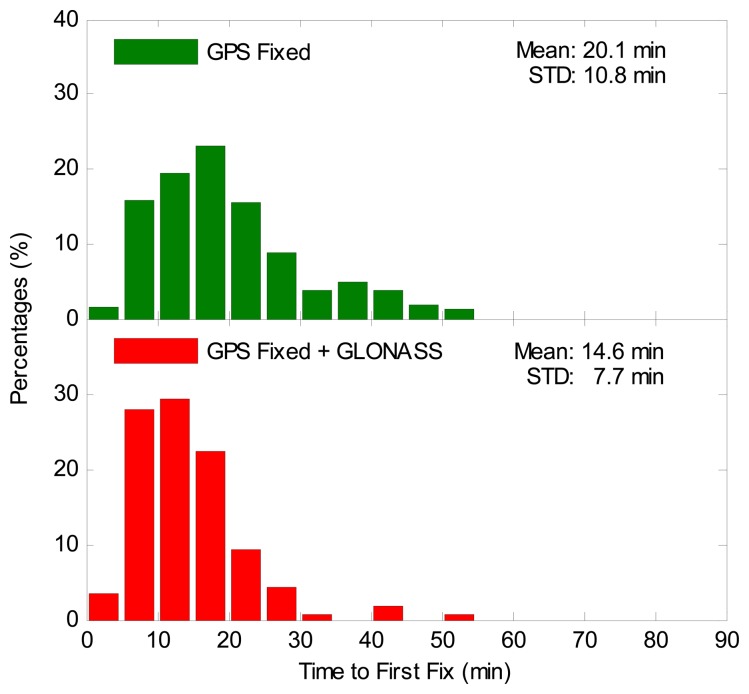
Distributions of time to first fix (TTFF) for GPS-only ambiguity-fixed PPP and GGPPP-FGA processing in eight sessions using datasets collected at 20 IGS stations on two consecutive days.

The TTFF is the period from the first epoch to the epoch when the first ambiguity-fixed solution is successfully obtained. The TTFF is different from the convergence time, since the positioning errors may still be larger than 0.1 m after the first-fixed solution. It is an important index to reflect the efficiency of achieving an ambiguity-fixed solution. Therefore, it is necessary to thoroughly assess the TTFF for the GPS-only case and the GPS/GLONASS case. Figure 10 illustrates the distributions of the TTFF. It is clearly seen that the TTFF for the GPS/GLONASS case is far smaller than that of the GPS-only case. The average TTFF for all sessions is also shown in Figure 10. The statistical results indicate that the average TTFF is reduced by 27% from 20.1 to 14.6 min after integrating GLONASS to GPS. The Wilcoxon rank sum test and chi-square two-sample test were also made for the TTFF. The Wilcoxon rank sum statistic value is 7.02, which is larger than the critical value of 1.96. The chi-square statistic value is 60.63, which exceeds the critical value, which is 18.31 with a DOF of 10. The statistical test results demonstrate that the improvement of the TTFF is significant for the GPS/GLONASS case over the GPS-only case.

The average number of satellites and position dilution of precision (PDOP) values for all three-hour sessions are computed. After adding GLONASS satellites, the number of satellites increases from an average of 7.7 to 13.5, leading to a decrease of PDOP values from 2.7 to 1.8. In general, the PPP ambiguity-float solutions can obtain higher accuracy with an increased number of satellites and an improved satellite geometry. As the accuracy of float ambiguity estimates has a strong positive impact on the successful rate and reliability of PPP IAR, it is easily understood why the performance of the GGPPP-FGA is better than the GPS-only ambiguity-fixed PPP.

## 5. Conclusions

A resolution for combined GPS/GLONASS precise point positioning (PPP) with fixed GPS ambiguities (GGPPP-FGA) is presented. Integrating GLONASS measurements with GPS can significantly improve the accuracy of GPS ambiguity-float estimates. Thus, the success rate and reliability of fixing GPS ambiguities can be enhanced. In the GGPPP-FGA, the ambiguity-float positioning solution is calculated firstly based on GPS singe-differenced (between satellites) and GLONASS un-differenced ionosphere-free measurements. Then, GPS ambiguities are fixed into integers, while all GLONASS ambiguities are kept as float values. An improved minimum constellation method (MCM) is proposed to enhance the efficiency of GPS ambiguity fixing.

Datasets from 20 globally distributed IGS stations on two consecutive days are employed to investigate the positioning accuracy, convergence time and the time to first fix (TTFF) for GPS-only ambiguity-float PPP, GPS-only ambiguity-fixed PPP and GGPPP-FGA. A total of 320 three-hour sessions are processed. The results indicate that the GGPPP-FGA achieves the best performance. The improvements of 43%, 8% and 43% on positioning accuracy and 47%, 6% and 50% on convergence time in the east, north and up coordinate components are achieved for GPS ambiguity-fixed PPP over GPS ambiguity-float PPP. Furthermore, the GGPPP-FGA improves the positioning accuracy by 38%, 25% and 44% and reduces the convergence time by 36%, 36% and 29% over GPS-only ambiguity-fixed resolutions in the three coordinate components, respectively. In addition, the TTFF is reduced by 27% after integrating GLONASS into the GPS ambiguity-fixed PPP. Moreover, Wilcoxon rank sum tests and chi-square two-sample tests are made to examine the significance of the performance improvement in terms of the positioning accuracy, convergence time and TTFF. The test results demonstrate that the performance improvement for the GGPPP-FGA over the GPS ambiguity-fixed PPP is significant in all three coordinate components. Numerical results indicate that the computation efficiency can be improved over 50% using the improved MCM algorithm.

Future work will include the investigation of the PPP integer ambiguity resolutions for multi-system combination of GPS, GLONASS, BeiDou and Galileo.

## Figures and Tables

**Table 1.** RMS statistics of CNES satellite orbit 3D errors and clock errors on 1 February 2014.

	GLONASS		GPS	
	
	Orbit (cm)	Clock (ns)	Orbit (cm)	Clock (ns)
ESA/ESOC	7.7	0.14	3.4	0.06
IAC	8.0	0.15	5.2	0.10
Mean	7.9	0.14	4.3	0.08

**Table 2.** Chi-square statistic values of positioning errors in the chi-square two-sample tests.

		GPS Fixed *vs.* GPS Float	GPS Fixed + GLO *vs.* GPS Fixed
East	$\chi_{0.05,\text{DOF}}^{2}$	30.14 (DOF = 19)	23.69 (DOF = 14)
	*χ*^2^	115.54	66.85

North	$\chi_{0.05,\text{DOF}}^{2}$	26.30 (DOF = 16)	21.03 (DOF = 12)
	*χ*^2^	70.90	23.74

Up	$\chi_{0.05,\text{DOF}}^{2}$	30.14 (DOF = 19)	25.00 (DOF = 15)
	*χ*^2^	117.92	105.99
